# The Olfactory System Revealed: Non-Invasive Mapping by using Constrained Spherical Deconvolution Tractography in Healthy Humans

**DOI:** 10.3389/fnana.2017.00032

**Published:** 2017-04-10

**Authors:** Demetrio Milardi, Alberto Cacciola, Alessandro Calamuneri, Maria F. Ghilardi, Fabrizia Caminiti, Filippo Cascio, Veronica Andronaco, Giuseppe Anastasi, Enricomaria Mormina, Alessandro Arrigo, Daniele Bruschetta, Angelo Quartarone

**Affiliations:** ^1^Centro Neurolesi Bonino Pulejo (IRCCS)Messina, Italy; ^2^Department of Biomedical, Dental Sciences and Morphological and Functional Images, University of MessinaMessina, Italy; ^3^Sophie Davis School for Biomedical Education, City College New York (CCNY), The City University of New York (CUNY)New York, NY, USA; ^4^The Fresco Institute for Parkinson’s and Movement Disorders, NYU Langone Medical Center, New York UniversityNew York, NY, USA; ^5^Department of Otorhinolaryngology, Papardo HospitalMessina, Italy

**Keywords:** olfactory system, olfactory tract, lateral stria, CSD, tractography

## Abstract

Although the olfactory sense has always been considered with less interest than the visual, auditive or somatic senses, it does plays a major role in our ordinary life, with important implication in dangerous situations or in social and emotional behaviors. Traditional Diffusion Tensor signal model and related tractography have been used in the past years to reconstruct the cranial nerves, including the olfactory nerve (ON). However, no supplementary information with regard to the pathways of the olfactory network have been provided. Here, by using the more advanced Constrained Spherical Deconvolution (CSD) diffusion model, we show for the first time *in vivo* and non-invasively that, in healthy humans, the olfactory system has a widely distributed anatomical network to several cortical regions as well as to many subcortical structures. Although the present study focuses on an healthy sample size, a similar approach could be applied in the near future to gain important insights with regard to the early involvement of olfaction in several neurodegenerative disorders.

## Introduction

The sense of smell plays a pivotal role in ordinary life. Together with the visual and auditory ones, it enables us to monitor the human environment and to provide an escape route from dangerous situations. More importantly olfaction has a big contribution in social and emotional behaviors, being able to make us revive old experience.

The anatomy of the olfactory system has been classically investigated by using microsurgery dissection (Kavoi and Jameela, 2011). More recently, olfactory tracts (OTs) were non-invasively studied by means of Diffusion Tensor Imaging (DTI) both in normal (Skorpil et al., 2011) and pathologic conditions (Scherfler et al., 2006). DTI fiber tracking has been extensively used to visualize cranial nerves (Hodaie et al., 2010), including the olfactory nerve (ON) and OTs. However, the intricate anatomy of the olfactory system (Price, 1990; Carmichael et al., 1994; Shipley and Ennis, 1996) and the presence of air in nasal sinuses makes DTI-based fiber tracking of OTs challenging. Despite those limitations, Skorpil and associates demonstrated for the first time, using a 1.5 T Magnetic Resonance Imaging (MRI) scan, that fiber tracking of the distal OTs is feasible (Skorpil et al., 2011).

Although DTI has been employed in several contexts (Basser and Pierpaoli, 1996; Pierpaoli and Basser, 1996; Pajevic and Pierpaoli, 1999; Le Bihan, 2003), it is unable to adequately characterize a system of fibers with complex axonal configurations (crossing, fanning, merging, bending and kissing; Tournier et al., 2007; Jones and Cercignani, 2010).

More sophisticated diffusion models, such as Constrained Spherical Deconvolution (CSD), permit to detect more consistently major fiber bundles in presence of intra-voxel orientational heterogeneity (Tournier et al., 2007, 2008). Indeed, CSD model allows to successfully identify multiple fiber populations insisting over the same voxel. CSD compares the signals observed in living tissues with a representative model of a single fiber bundle, the so-called “response function”. By means a mathematical process, the spherical deconvolution, CSD attempts to directly estimate from the Diffusion Signals (DWs) the so-called fiber Orientation Distribution Function (fODF), i.e., an approximation of the distinct fiber directions that insist over white matter voxels CSD has been shown to resolve most of typical DTI model limitations (Tournier et al., 2007, 2008).

Using this approach, we have recently shown that a consistent reconstruction of the basal ganglia, red nucleus, claustrum and limbic connectome is feasible (Arrigo et al., 2014; Milardi et al., 2015a,b, 2016a,b; Mormina et al., 2015; Cacciola et al., 2016, 2017).

Therefore, by taking advantage of CSD diffusion model, the primary aim of this article was to provide an anatomical *in vivo* reconstruction of both the short and long direct fiber pathways of the olfactory system. Neurophysiological and clinical considerations are widely discussed.

## Materials and Methods

### Participants

A total of 10 healthy subjects (6 males, 4 females; mean age 32.1; age range 25–50 years) without any overt neurological, psychiatric or traumatic disease were recruited. The entire study was approved by Institutional Review Board of IRCCS Bonino Pulejo—Messina—Italy (Scientific Institute for Research, Hospitalization and Health Care) and all subjects received and signed an inform consent before MRI examination.

### Data Acquisition

The study was performed with a 3-T Achieva Philips equipped with a 32-channel SENSE head coil (Best, Netherlands). For each subject, the following MRI protocol was carried out:

- (3D) high-resolution T1-weighted fast field echo (FFE) sequence, with the following parameters: Repetition Time 25 ms; Echo Time 4.6 ms; flip angle 30°; Field of View (FOV) 240 × 240 mm^2^; reconstruction matrix 240 × 240; voxel size 1 × 1 × 1 mm. Total acquisition time was 6 min.
- A single-shot echo-planar Imaging (SS-EPI) diffusion weighted sequence, with the following parameters: Repetition Time 11884 ms; Echo Time 54 ms; FOV 240 × 240 mm^2^; scan matrix 120 × 120; in-plane resolution 2 × 2 mm, axial slice thickness 2 mm without inter-slice gap. One unweighted b0 volume and 30 diffusion encoding directions (*b*-value = 1000 s/mm^2^) covering a half sphere were acquired following the rules stated by an electrostatic repulsion model (Jones et al., 1999). The total acquisition time was 9 min. In addition, a b0 volume was acquired by using a reverse phase-enconding direction for post-acquisition artifacts correction.

### Data Processing, Segmentation and Tractography

Diffusion data were corrected for motion and susceptibility artifacts using top-up and eddy FMRIB Software Library (FSL) tools (Jenkinson et al., 2002,^1^). Rotational part of transformation was applied to gradient directions at the end of this stage.

To model the Diffusion Signal (DW), we used a modified High Angular Resolution Diffusion Imaging (HARDI) technique, called nonnegative CSD. This model estimates the so-called fODF by deconvolving the DW signal with a representative single fiber response function (Tournier et al., 2007). By using CSD, we managed to overcome poor representation of complex fiber geometries typical of DTI (Tournier et al., 2008). We reconstructed a color-coded map in which red (left-right), blue (inferior-superior) and green (anterior-posterior) colors indicate the principal eigenvector’s direction (Pajevic and Pierpaoli, 1999). Response function estimation and CSD fitting were performed by using MRtrix software, release 3 (Tournier et al., 2012,^2^).

**Figure 1:**
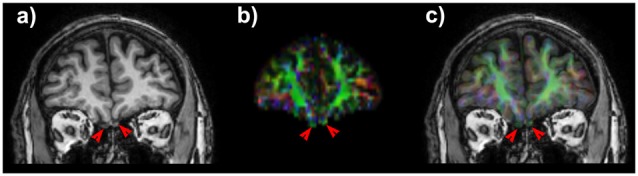
**Coronal T1 weighted image shows the olfactory tracts (OTs). (A)** Note the slight positional asymmetry on the cranio-caudal plane. On anisotropic map **(B)** OTs appears green due to their main antero-posterior direction. A good matching between T1 weighted scan and diffusion maps **(C)** is necessary to obtain consistent and robust results for of such small anatomic structures.

Structural T1w volumes were co-registered to diffusion data by means of a non-linear procedure based on cerebrospinal fluid (CSF) probability maps coming from T1w and b0 images. Details of the pipeline are reported in Besson et al. (2014). Estimation of CSF probability maps was performed by means of New Segment command provided within the Matlab based Statistical Parametric Mapping tool, release 8 (SPM8). Non-linear registration was accomplished by means of FSL flirt and fnirt routines. Segmentation of the OTs was manually performed on co-registered T1-weighted structural scans by an experienced neuroradiologist. An overlap as accurate as possible between diffusion data and structural maps (Figure 1) is crucial since tractographic reconstruction could be spoiled due to well-known asymmetry in the cranio-caudal position of the tracts, their small size and the presence of air in the ethmoid cells. The pyriform cortex was firstly identified in a oblique-axial section with +20° relative to the anterior commissure-posterior commissure axis thus allowing to see the relationship between the piriform cortex, amygdala and hippocampus (Vaughan and Jackson, 2014). Taking into account that there are no clues to outline the frontal borders of the pyriform cortex and that its frontal portion represent only the 10%–15% of the total pyriform cortex volume on MRI, we focused on its temporal extension (Gonçalves Pereira et al., 2005).

The Desikan-Killiany atlas included in the FreeSurfer image analysis suite (Fischl et al., 2004, available at http://surfer.Nmr.mgh.harvard.edu/) was employed to obtain volumetric segmentation of the amygdala and entorhinal cortex based on the co-registered T1 images. Successively, the segmentations obtained from each individual were visually inspected and, if needed, manually edited. In addition, we segmented the fornix and optic system from the optic nerve up to the lateral geniculate nucleus to avoid the reconstruction of spurious streamlines running through such regions.

Fiber-tracking was performed using Mrtrix package (Tournier et al., 2012), release 3. The step size of the tracking algorithm was set at 0.2 mm, the minimum radius of curvature at 1 mm and the fODF amplitude cutoff for terminating tracks was 0.15. Multiple Regions of Interest (ROIs) as well as Regions of Avoidance (ROAs) were combined to fulfill the complicated tracking demand for each fiber bundle reconstruction (Verstynen et al., 2011).

## Results

In all subjects we obtained an accurate co-registration of T1 weighted scan and anisotropic map (Figure 1) and we were able to reconstruct several white matter bundles forming the olfactory network.

### Lateral Striae

**Figure 2:**
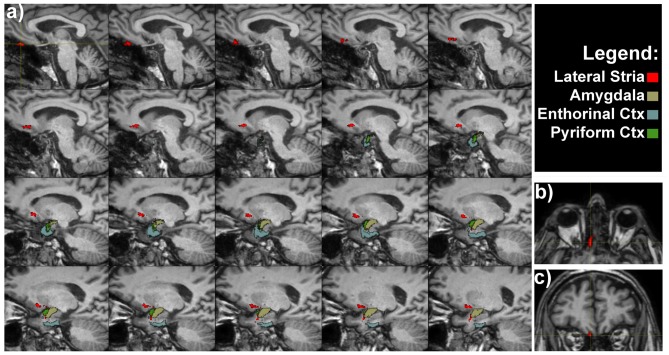
**Olfactory lateral stria of a representative subject. (A)** Medio-lateral sagittal views show the course of OT connections with amygdala, entorhinal and pyriform cortices. Axial **(B)** and coronal **(C)** views of the OT, as indicated by the crosshair and red voxels.

OTs projected directly to the piriform cortex and to the olfactory tubercle running through the anterior perforated substance. Olfactory tubercle is very small and difficult to be detected at the resolution used in this study, therefore its segmentation was not carried out (Porter et al., 2005). We detected a pathway connecting OT and the rostral temporal lobe in the piriform cortex (Figure 2).

The OTs also projected to the rostromedial temporal lobe in the parahippocampal gyrus, where the rostral entorhinal cortex is located (Figure 2). In addition direct projections between the amygdala and OT were detected (Figure 2). The entire course of the lateral striae in coronal view is shown in Supplementary Figure S1.

### Olfactory Direct and Indirect Pathways

**Figure 3:**
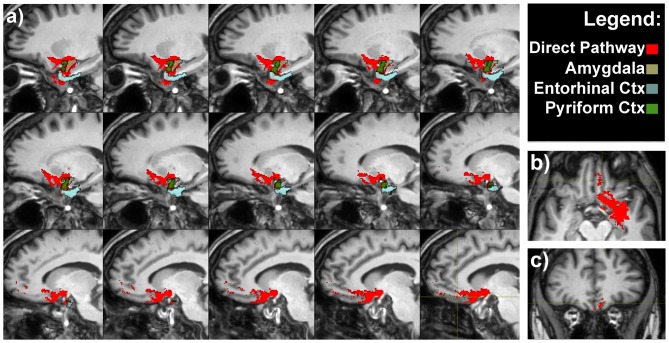
**Olfactory direct pathway. (A)** Latero-medial sagittal views of the connections between the primary olfactory cortices—amygdala, entorhinal and piriform cortices—with the medial orbitofrontal cortex (OFC). Axial **(B)** and coronal **(C)** representative views of the same pathways.

A bundle directly connecting piriform lobe with orbitofrontal cortex (OFC) could be seen. The fibers surrounded the temporal horn of the lateral ventricle and run through the sublenticular white matter (Figure 3).

It is worth to note that pathways 1, 2 and 3 reached the piriform cortex, enthorinal cortex and amygdala which in turn were reciprocally connected to the medial OFC via the direct olfactory pathway (Figure 3 and Supplementary Figure S2).

**Figure 4:**
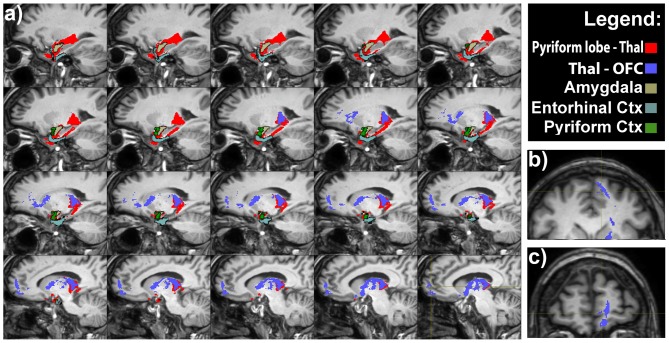
**Olfactory indirect pathway. (A)** Latero-medial sagittal views of the connections between the pyriform lobe and the MDTN (in red), and between medio-dorsal nucleus of the thalamus (MDNT) and the medial OFC. Axial **(B)** and coronal **(C)** views of the indirect pathway at the level of the medial OFC.

An indirect projection via the medio-dorsal nucleus of the thalamus (MDNT) has been previously demonstrated (Ongür and Price, 2000). However, we could not reconstruct such indirect disynaptic pathway due to the inability of tractography reconstruction to provide definitive information on multisynaptic connections passing through more than two gray matter structures. On the other hand, we demonstrated the presence of connections running between the pyriform lobe and the MDNT and the MDNT and the OFC. Therefore, based on this finding, we can speculate that these connections may represent the anatomical substrate of the olfactory indirect pathway (Figure 4).

## Discussion

By using CSD diffusion model we showed for the first time *in vivo* that, in normal humans, olfactory system has a widely distributed anatomical network to several cortical regions as well as to many subcortical structures. These data extend previous works showing that CSD is a robust technique for the *in vivo* reconstruction of the intricate and complex brain networks (Lambert et al., 2012; Arrigo et al., 2014; Milardi et al., 2015a,b, 2016a,b; Mormina et al., 2015; Cacciola et al., 2016, 2017).

To the best of our knowledge, so far only one article has used DTI for studying the olfactory system (Skorpil et al., 2011). More in detail, Skorpil et al. (2011) performed DTI-based tractographic reconstruction on five patients with a 1.5 T to reconstruct OTs; however, no supplementary information of the other bundles of the olfactory network were provided. In addition, few data exist on DTI abnormalities in pathologic conditions (Scherfler et al., 2006, 2013; Erb et al., 2012; Erb-Eigner et al., 2014).

As outlined in the “Introduction” Section, DTI is unable to identify multiple fiber regions within a voxel; it is however known that more than 90% of white matter voxels have complex structures (Jeurissen et al., 2013). Therefore, it is inadequate in most occasions. CSD attempts to resolve multiple fiber geometries by comparing observed signals with a representative single fiber “response function”. Unlike DTI, CSD overcome most of DTI limitations, such as the partial volume effect (Jones and Cercignani, 2010), thus resulting in improved tractographic performances. Other models exist to provide more useful insights into white matter structures than what DTI is able to do, like for instance Q-ball imaging (QBI, Tuch, 2004), or Diffusion Spectrum Imaging (DSI, Wedeen et al., 2008). Nevertheless, for a clinical use, DSI is less used since it requires longer scan time (Tournier et al., 2007), whilst QBI suffers from technical restraints, such as the inability to reconstruct fibers with crossing angle smaller than 45° (Tournier et al., 2008; Gigandet et al., 2013). Taking into account the abovementioned considerations, probabilistic CSD is to date, probably, the most promising approach for tractographic reconstruction in a clinical context.

### Olfactory Tract: Lateral and Medial Striae

The olfactory bulb (OB) sends direct projections to the anterior olfactory nucleus which in turn projects back to the ipsilateral and contralateral OB via the anterior commisure and to several secondary olfactory areas (Mohedano-Moriano et al., 2012). The OT is described as a band of white fiber matters originating from tufted and mitral cells within the OB that splits into medial and lateral striae, before reaching the perforate substance (PF; Duprez and Rombaux, 2010). Furthermore, tract-tracing studies in primates have demonstrated that the anterior olfactory nucleus is connected to orbitofrontal areas 12 and 13, which are both functionally and anatomically related to olfactory processing (Mohedano-Moriano et al., 2005). In contrast to anatomical data, we were not able to obtain consistent tractographic CSD-based reconstructions linking the OT and anterior olfactory nucleus to the contralateral OT via the anterior commissure (Risse et al., 1978).

In particular, the presence of air in ethmoidal cells may interfere with EPI sequences as already reported in literature (Skorpil et al., 2011). On the other hand, we could detect the different portions of the lateral olfactory stria projecting directly (Smythies, 1997; Gottfried and Zald, 2005; Wilson and Sullivan, 2011) to piriform and entorhinal cortices and amygdala (Price, 1985, 2003; Porter et al., 2005) without thalamic relay. Positron emission tomography studies combined with olfactory stimulation showed significant cerebral blood flow increase in the bilateral pirifom cortex, the rostromedial region of the entorhinal cortex and in the right OFC (Zatorre et al., 1992). The major role of the piriform cortex is to discern odors of different categories: more in detail the anterior piriform cortex is correlated with odor identification, whilst the posterior one is correlated with odor categorization (Gottfried et al., 2006; Wilson et al., 2006; Howard et al., 2009). On the other hand, it has been suggested that the rostromedial area of the entorhinal cortex sends topographically organized projections to the temporal region of the hippocampus, thus partially explaining the presence of “olfactory auras” and “olfactory hallucinations” in many neurological disorders (Insausti et al., 2002).

### Olfactory Direct and Indirect Pathway to Neocortex

Odorant information is transmitted from the olfactory cortices directly to the OFC and via the thalamus (direct and indirect pathways).

The indirect pathway through the thalamus is thought to account for the perceptions and discriminations of odors, because people with extensive damage of the OFC are unable to identify (Jones-Gotman and Zatorre, 1998) and to discriminate odors (Zatorre and Jones-Gotman, 1991; Caminiti et al., 2013). As pointed out before, the main characteristic of the olfactory system is the absence of primary connections with the thalamus; since the axons of OBs neurons project directly to the primary olfactory cortex (Smythies, 1997; Spence et al., 2001b; Gottfried and Zald, 2005; Murakami et al., 2005; Shepherd, 2005). In this study we showed for the first time that olfaction has a direct route to the neocortex connecting the piriform, entorhinal cortices and amygdala to the OFC; those findings are in keeping with an established body of research based on both humans and animals (Takagi, 1986; Price et al., 1991; Johnson and Leon, 2000).

In addition, we found tractographic evidences of direct connections running between the pyriform lobe and the MDNT and the MDNT and the OFC. As suggested in the previous section, we speculate that these connections may represent the anatomical substrate of the olfactory indirect pathway.

### Physiological and Clinical Considerations

Although the olfactory sense has always been considered with less interest than the visual, auditive or somatic senses, it does plays a major role in our ordinary life. A proof of concept is that despite the smell in humans is less important than in other species, in which the odor detection is essential for searching for food, recognition of the presence of predators or for sexual coupling (Manzini et al., 2014), nonetheless the structure of the human olfactory system is extremely sophisticated.

Indeed, olfaction has some positive physiological cross-modal influence on several behavioral domains such as attention (Spence et al., 2001a), emotion (Herz et al., 2004), memory (Herz, 1998), airflow motor control (Bensafi et al., 2003), scent tracking (Porter et al., 2006) and visuo-motor interactions (Castiello et al., 2006).

The prerogative of different odors to evoke pleasant or unpleasant sensations implies that the olfactory sensitivity may affect individual’s emotional reaction to the environment, person or object wearing a specific odor (Rolls et al., 2003). This is particularly important in determining the palatability of a food or a drink, emphasizing the involvement of smell in the regulation of food intake. It is also noteworthy how certain odors remain long as a component of the involuntary memory of pleasant or unpleasant events, and how a certain smell can bring out the same emotions produced by the first experience of it; a phenomenon similar of that described by Proust and correlated with the Madeleine’s cake taste (Proust, 1987–89). The “memory of odors” has also long-term effects on reflexes activity: a typical example is the nausea associated with the smell of food, which has caused vomiting in the past. Some of these considerations are strongly supported by the existence of the intricate olfactory network demonstrated in the present study (Figures 2–4).

It has been demonstrated that the piriform cortex and the olfactory tubercle are strictly connected to the MDTN via the so-called indirect pathway (Ongür and Price, 2000). This circuit along with the direct circuit, which project from the pyriform lobe to orbito-frontal cortex, is involved in the conscious perception of odors (Zald and Rauch, 2008). Hence, both the olfactory direct and indirect pathways reconstructed in the present study, by using CSD-based tractography, should be further investigated in combined MRI and psychophysiological studies to shed new light on their functions.

Lesion studies in rats have shown that MDNT lesions might led to several olfactory functions impairment such as olfactory discrimination (Eichenbaum et al., 1980), odor reversal learning (McBride and Slotnick, 1997) and odor-guided sexual behavior (Sapolsky and Eichenbaum, 1980).

In a clinical context, studies on patients with Korsakoff’s syndrome as well as with focal lesions of the MDNT further suggest a significant role of MDNT in olfactory function. On the other hand, given the wide neural impairment in Korsakoff’s syndrome (Victor et al., 1971; Ridley et al., 2005), and the occurrence of cases of Korsakoff’s without detectable damage to MDNT (Mair et al., 1979), it is difficult to pinpoint the precise role of the MDNT in olfaction from these patients alone.

Sela et al. (2009) tested 17 patients with unilateral focal thalamic lesions and 18 age-matched controls on a battery of tests that included olfactory detection, olfactory identification, and olfactory pleasantness estimation. These investigators found that thalamic lesions did not affect olfactory detection but significantly impaired olfactory identification, and only right lesions altered olfactory hedonics of pleasant odors.

Finally, in a recent psychophysiological study, it has been demonstrated that patients with MDNT lesions had varying impairments in the olfactory attention domain but not on most general olfactory ability, thus suggesting that the MDNT pathway plays, either a specific or generic, role in mediating olfactory attention in humans (Tham et al., 2011).

In addition, olfactory memory relies on the connections with entorhinal and piriform cortices, which are exchanging extensive information with hippocampus (Price, 1990; Kerr et al., 2007).

Finally the associations of odors to emotional stimuli is supported by the direct connections of OT to amygdala and from the reciprocal connections between pyriform and entorhinal cortex as described in animal studies.

It is worthy to note that olfactory abnormalities are present in a wide range of neurological disorders such as, Multiple Sclerosis (Rolet et al., 2013), Huntington disease (HD; Barrios et al., 2007), Alzheimer disease (Ferreyra-Moyano and Barragan, 1989) and Parkinson’s disease (PD; Doty et al., 1988; Hawkes et al., 1997, 1999).

Impairment in the sense of smell is often associated with PD, probably due to hippocampal dopaminergic denervation (Bohnen et al., 2008). The olfactory deficits typically occur very early in the disease process, long before the damage in the basal ganglia and the onset of motor-related symptoms (Braak et al., 2004). It is well known that according to the Braak stage, alpha-synuclein accumulation initially occurs in the OBs and the anterior olfactory nucleus (in addition to the dorsal motor nuclei of the IX and X cranial nerves) thus leading to prodromal non-motor symptoms such as hyposmia. In addition, it is likely that central olfactory structures such as the entorhinal and piriform cortices are involved in the third stage of the disease (Braak et al., 2003). However, it remains still unclear to what extent alpha-synuclein accumulation and dopamine receptors dysfunctions both in peripheral and central olfactory structures contribute to the etiopathology of PD (Ubeda-Bañon et al., 2014). In a combined [^123^I]β-CIT-SPECT study, Berendse et al. (2001) have shown a sub-clinical abnormal reduction in striatal dopamine transporter binding in asymptomatic PD patients’ relatives with quantitative olfactory dysfunction (Berendse et al., 2001). In line with this finding, it is likely that relative of PD patients with olfactory deficit have a 10% chance of developing clinically manifest PD (Ponsen et al., 2004). In addition, several PET and SPECT imaging studies in early-stage PD patients have demonstrated correlations between olfactory test scores and dopamine transporter levels in the striatum and hippocampus (Siderowf et al., 2005; Bohnen et al., 2008).

These findings further suggest that olfactory deficits may precede clinical motor signs of PD and further studies should be fostered to evaluate olfactory involvement as early biomarker of the disease. In this perspective, CSD-based analysis may help to provide robust reconstruction of olfactory system in order to detect PD related symptoms at a very early stage.

Recently, Rolheiser et al. (2011) revealed significant differences of FA values in the substantia nigra and anterior olfactory region, as the OT, between normal and PD subjects, in a combined study of olfactory testing and DTI. Moreover, FA reduction has also been reported in PD in a voxel-based DTI study (Ibarretxe-Bilbao et al., 2010). Particularly, they showed lower FA values in the primary olfactory cortex (piriform cortex, the anterior cortical nucleus of the amygdala and the rostral entorhinal cortex), associated with reduced smell sensation (Ibarretxe-Bilbao et al., 2010).

In addition, it has been demonstrated that olfactory function is compromised in early stages of AD. Although the pathophysiology of olfactory impairments in AD is still not clear, it has been suggested that pathological accumulation of tau protein in the OBs and olfactory related areas may play a key role for the changes in olfactory identification, recognition and olfactory detection threshold (Braak et al., 1993; Attems et al., 2005). In line with this hypothesis, by using olfactory fMRI, Wang et al. (2010) detected olfactory deficits in AD, showing significant reduced activation in several olfactory areas such as the primary olfactory cortices, the thalamus, hypothalamus and hippocampus (Wang et al., 2010).

Early odor dysfunctions have been documented in HD as well: indeed, odor memory impairment have been found to occur before the onset of cognitive deficit and involuntary movements (Moberg et al., 1987). On the other hand, several authors have shown that HD patients had significant deficits in the odor identification domain, but not in odor recognition memory (Bacon Moore et al., 1999; Nordin et al., 1995). In a voxel-based morphology study in patients with HD, it has been revealed significant volume reductions in olfactory-related areas such as the gyrus rectus and the parahippocampal gyrus, in addition to a significant correlation between degeneration of the entorhinal cortex, the caudate and thalamic nuclei, parahippocampal gyrus and the olfactory psychophysical deficit (Barrios et al., 2007). Concurrently to these findings, knock-in mouse models of HD with 140 CAG repeats have shown that early accumulation of huntingtin protein containing aggregates occurs in the olfactory system (Menalled et al., 2003).

Altogether, these findings suggest the need to further develop new approaches for the investigation of the olfactory system in patients with neurodegenerative disease both in the early and later stages of the pathology. Our results showed that the use of CSD-based tractography may provide a new framework to investigate olfactory dysfunctions and brain connectivity of the olfactory network in patients with neurodegenerative disorders.

## Limitations of the Study

The present study is not prone of limitations. A technical intrinsic weakness of tractography is the incapability to detect the presence of synapsis and gap junctions as well as the directionality (afferent-efferent) of the signal transmission (Chung et al., 2011; Parker et al., 2013; Milardi et al., 2015b).

Tractography is rather a technique which provides the likelihood of connection between two given anatomical areas, thus giving only an indirect measure of the underlying potential existence of an anatomical pathway and cannot be taken as definitive evidence for either pathway.

In keeping with previous findings of our group, we confirmed however that CSD-based tractography is a valuable technique which allows a robust reconstruction of both short and long white matter pathways in brain regions presenting fibers with complex geometry (Kristo et al., 2013; Arrigo et al., 2014; Milardi et al., 2015a; Mormina et al., 2015; Cacciola et al., 2017) using 3T MRI scanners. This approach can overcome most of the DTI limitations including those related to low anisotropy crossing fibers (Parker et al., 2013).

We were unable to detect and reconstruct the proximal part of the OTs. This limitation may be due to: (i) the tracts splitting up into very thin diameters fibers compared to the diffusion weighted axial slice thickness and voxel resolution; and (ii) to well-known susceptibility artifacts that are inherent to the area of interest (presence of air in ethmoidal cells) in EPI sequences.

## Conclusion

Despite the above-mentioned limitations, our work provided anatomical *in vivo* reconstruction of the extensive olfactory network by using CSD diffusion model. Such non-invasive mapping should be further improved with higher spatial resolution imaging in order to provide not only a qualitative assessment of the olfactory system, but also a quantitative evaluation of diffusion parameters. A similar approach could be used in the near future to explore the early involvement of olfaction in several neurodegenerative disorders.

## Author Contributions

DM: study concepts/study design, data acquisition, data analysis, data interpretation, literature research. AlbC: Study concepts/study design, data acquisition, data analysis, data interpretation, literature research, draft the work. AleC: study concepts/study design, data acquisition, data analysis, manuscript revision. MFG: data interpretation, manuscript revision for important intellectual content. FaC and FiC: data acquisition, data interpretation, literature research. VA: data analysis, data interpretation, literature research. GA: guarantor of integrity of entire study, data interpretation, manuscript revision for important intellectual content. EM, DB and AA: data analysis, literature research. AQ: study concepts/study design, guarantor of integrity of entire study, manuscript revision for important intellectual content. All the authors approved the final version of the manuscript.

## Conflict of Interest Statement

The authors declare that the research was conducted in the absence of any commercial or financial relationships that could be construed as a potential conflict of interest.

## Footnotes

^1^https://www.fmrib.ox.ac.uk/fsl

^2^www.mrtrix.org

## Supplementary Material

FIGURE S1 **Olfactory lateral stria.** Sagittal **(A)** and axial **(B)** views of the proximal portion of the lateral stria. Coronal sections **(C)** show the entire course of the right and left lateral striae, from the olfactory tracts (OTs) to the amygdala, entorhinal and pyriform cortices.

FIGURE S2 **Olfactory direct pathway.** Sagittal **(A)** and axial **(B)** views of the pathway at the level of the medial orbitofrontal cortex (OFC). Coronal sections **(C)** show the entire course of the right and left olfactory direct pathway connecting the amygdala, entorhinal and piriform cortices with the medial OFC.
