# Supplementary figures and images for: The Olfactory System Revealed: Non-Invasive Mapping by using Constrained Spherical Deconvolution Tractography in Healthy Humans
Source: Front Neuroanat. 2017 Apr 10;11:32. doi: 10.3389/fnana.2017.00032 (PMC5385345; doi:10.3389/fnana.2017.00032)

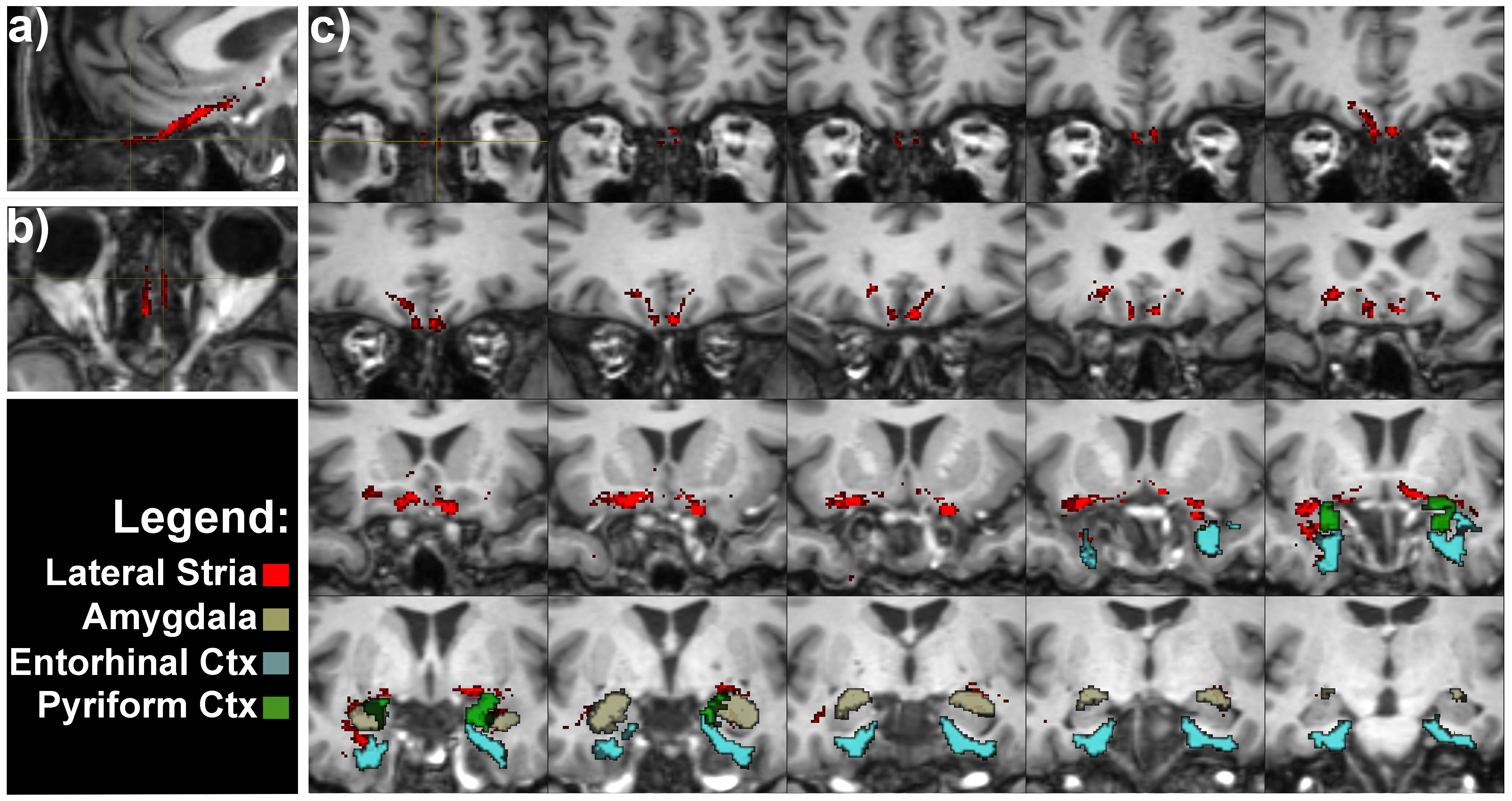

Supplement: FIGURE S1 — Olfactory lateral stria. Sagittal (A) and axial (B) views of the proximal portion of the lateral stria. Coronal sections (C) show the entire course of the right and left lateral striae, from the olfactory tracts (OTs) to the amygdala, entorhinal and pyriform cortices. [file Image_1.tif]

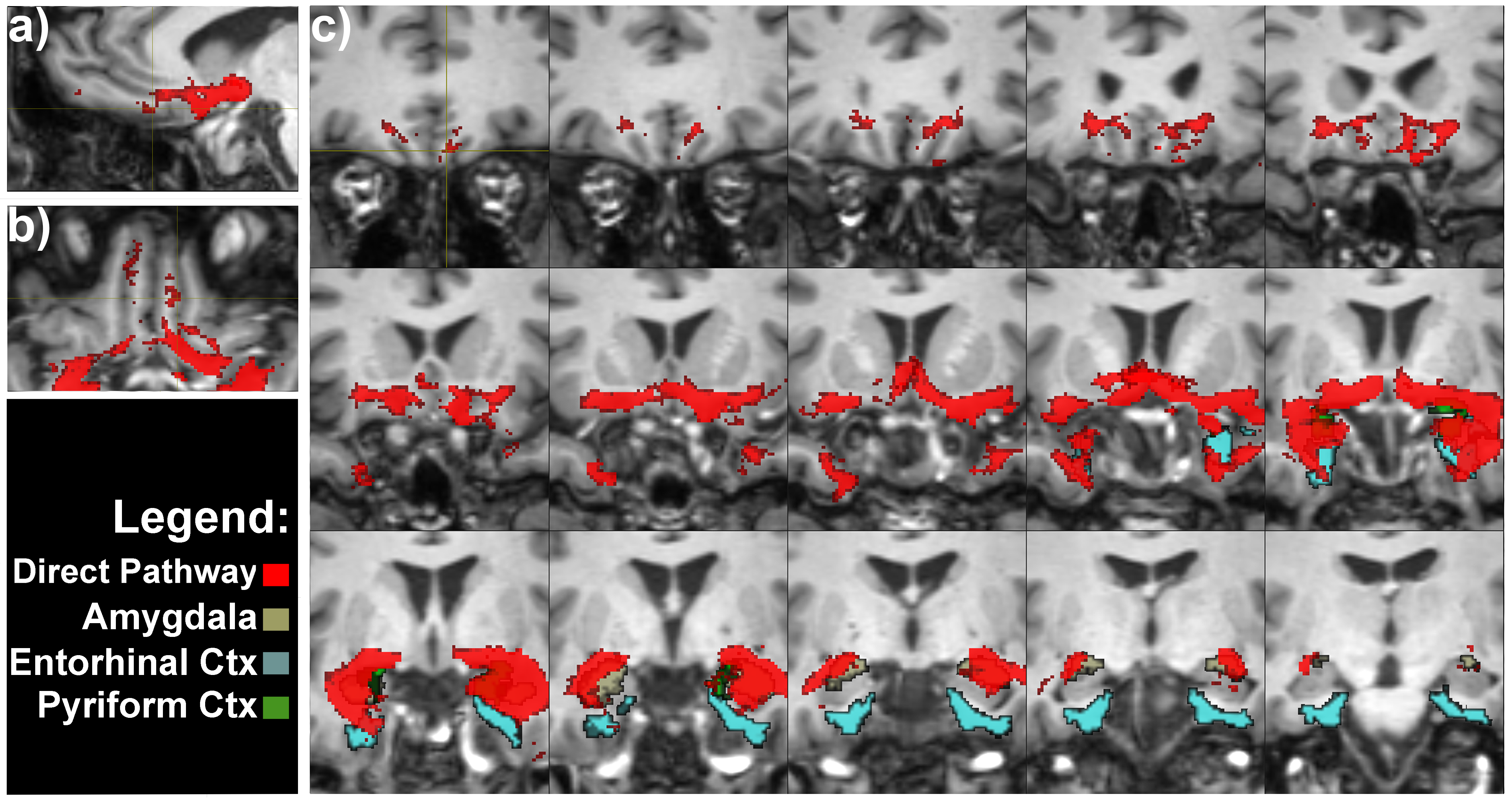

Supplement: FIGURE S2 — Olfactory direct pathway. Sagittal (A) and axial (B) views of the pathway at the level of the medial orbitofrontal cortex (OFC). Coronal sections (C) show the entire course of the right and left olfactory direct pathway connecting the amygdala, entorhinal and piriform cortices with the medial OFC. [file Image_2.tif]
